# One-pot multicomponent green Hantzsch synthesis of 1,2-dihydropyridine derivatives with antiproliferative activity

**DOI:** 10.3762/bjoc.16.235

**Published:** 2020-11-24

**Authors:** Giovanna Bosica, Kaylie Demanuele, José M Padrón, Adrián Puerta

**Affiliations:** 1 Department of Chemistry, University of Malta, Msida, MSD 2080 Malta; 2 BioLab, Instituto Universitario de Bio-Orgánica “Antonio González” (IUBO-AG), Universidad de La Laguna, c/Astrofísico Francisco Sánchez 2, 38206 La Laguna, Spain

**Keywords:** antiproliferative activity, 1,2-dihydropyridines, green Hantzsch synthesis, heterogeneous catalysis, one-pot multicomponent reaction

## Abstract

A rapid route for obtaining unsymmetrical 1,2-dihydropyridines (1,2-DHPs) as opposed to 1,4-dihydropyridines (1,4-DHPs) has been achieved via a one-pot multicomponent Hantzsch reaction. A benign protocol has been developed for the preparation of various 1,2-dihydropyridine derivatives using heterogenized phosphotungstic acid on alumina support (40 wt %). High yields of over 75% have been accomplished in just 2–3.5 h after screening several heterogeneous catalysts and investigating the optimal reaction conditions. The catalyst chosen has passed the heterogeneity test and was shown to have the potential of being reused for up to 8 consecutive cycles before having a significant loss in activity. In addition, aromatic aldehydes gave the aforementioned regioisomer while the classical 1,4-DHPs were obtained when carrying out the reaction using aliphatic aldehydes. The preliminary study of the antiproliferative activity against human solid tumor cells demonstrated that 1,2-DHPs could inhibit cancer cell growth in the low micromolar range.

## Introduction

A multicomponent approach towards the synthesis of the desired product offers a number of advantages over a stepwise method. Such advantages include the development of a design that is: cheaper, simpler, economical, and environmentally friendly [1–2]. Multicomponent reactions are not new to research. The pioneer multicomponent reactions are the Hantzsch (1882), Biginelli (1891), Mannich (1912), Passerini (1921), and Ugi (1959) reactions [3]. The significance of such a phenomenal approach for the synthesis of novel compounds first began as a way of increasing the chemical libraries and then shifted to obtaining products that are in high demand on an industrial scale at a cheaper and more benign way [4]. Recently, negative human impacts have been greatly witnessed as a result of population growth, so environmentally friendly design has become one of the most important contributions. As a result, such research has grown exponentially in the past decade [5–6].

The work conducted by the German chemist Arthur Hantzsch exploded in the synthetic interest in dihydropyridines and pyridines when the pharmacological usefulness of these compounds in medicine was discovered [7]. The structural resemblance of these compounds to the coenzyme reduced nicotinamide adenine dinucleotide (NADH) sparked the potential pharmaceutical properties, and till today, the Hantzsch synthesis is the main route for obtaining such products, which are eventually used as active pharmaceutical ingredients in the pharmaceutical industry [3,8]. An analysis of the market shows that there are over 7000 drugs derived from dihydropyridines, some of which are blockbuster drugs, such as Tamiflu^®^, dioscorine, ibogaine, and isoquinuclidines [9–10].

**Scheme 1:**
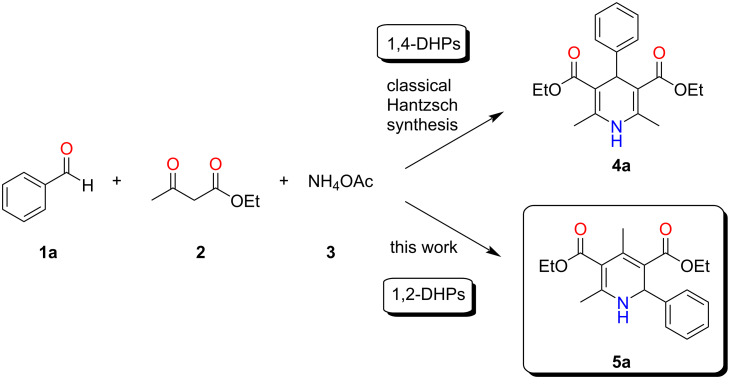
The classical Hantzsch synthesis between benzaldehyde (**1a**), ethyl acetoacetate (**2**), and ammonium acetate (**3**) as well as the synthesis highlighted in this work.

Classically the Hantzsch synthesis involved the condensation of 2 equivalents of the β-ketoester ethyl acetoacetate (**2**) with benzaldehyde (**1a**) and ammonia (Scheme 1) [11]. This procedure was later optimized over the years using different substrates by varying the β-ketoesters and aldehydes in order to prepare a larger array of 1,4-dihydropyridines (1,4-DHPs) [12]. In addition, further developments were made to the methodology in order to enhance the reaction yield and also to reduce the reaction time. Other recent developments involve reducing the energy and waste that is produced in the reaction for a more environmentally friendly synthesis [13–16]. The source of nitrogen has also been varied from the classical use of ammonia. The most common nitrogen source reported in literature is ammonium acetate (**3**). Others include the use of oxahydrazines, primary amines, and urea. The oxidation of dihydropyridines to pyridines has been achieved using mild oxidizing agents [7,17–18].

One of the greatest limitations of this synthesis is however the fact that the dihydropyridines that are obtained are usually the 1,4-symmetrical ones. This multicomponent reaction has been thought to have one of the most complex mechanisms since various routes might take place, and the mechanism depends much on the identity of the substrates and the reaction conditions used [18]. Cao and collaborators have managed to synthesize the 1,2-dihydropyridine (1,2-DHP) regioisomer as the main product through the Hantzsch synthesis at room temperature and solvent-free conditions, irrespective of the electronic effect of the substituted benzaldehydes studied [19]. This was a further improvement of the reaction since the usual regioisomer has always been reported to be the 1,4-DHP. Cao et al. have suggested an alternative mechanism for this route. When the same reaction was conducted under argon, they obtained a mixture of the two regioisomers (1,4-DHP/1,2-DHP 32:68), proving further the complexity of this reaction [19].

Therefore, continuing our studies for the development and application of environmentally friendly methodologies for multicomponent reactions [20], we attempted to find a green catalyst that could provide a wide substrate scope for the Hantzsch synthesis of 1,2-dihydropyridines in a short reaction time.

In order to achieve a green method, apart from utilizing a multicomponent reaction as a route providing a high atom economy, heterogeneous catalysis should be used since it offers a greener alternative to homogeneous catalysis and ideally a solvent-free design to reduce the amount of solvent waste [21–22]. These two factors will reduce the amount of hazardous chemicals by reducing the amount of solvent in the reactor and during the workup of the product. A solid insoluble catalyst can easily be removed from the reaction mixture via filtration, unlike a soluble one [23–25].

## Results and Discussion

According to literature, the reaction has shown to work best and most efficiently under acidic conditions since such conditions enhance the selectivity. When the model reaction between benzaldehyde (**1a**), ethyl acetoacetate (**2**), and ammonium acetate (**3**, Scheme 1) was carried out in the absence of any catalyst, it turned out to be very slow and, according to GC chromatograms, stopped in the early stages since the peaks of the corresponding starting materials of the model reaction appeared. A number of acidic catalysts was then analyzed before choosing the optimal catalyst (Table 1). The most significant result selected was based on the yield and reaction time.

**Table 1:** Screening of acidic heterogeneous catalysts.

entry^a^	catalyst	yield (%)^b^	reaction time (h)

1	Nafion^®^ NR-50	88	5
2	Nafion^®^ SAC-13	96	5
3	montmorillonite K30	72	4.5
4	Dowex^®^ 50W	48	5.5
5	Amberlyst^®^ 15	68	5.5
6^c^	activated Amberlyst^®^ 15	82	5
7	40 wt % silicotungtstic acid on cellulose	49	6
8	40 wt % silicotungstic acid/Al_2_O_3_	37	6
9	40 wt % phosphotungstic acid (PW)/SiO_2_	61	6
10	20 wt % silicotungstic acid/montmorillonite K10	40	6
11^d^	30 wt % PW/montmorillonite K30	83	2.5
12	40 wt % PW/Al_2_O_3_	94	3.5
13	40 wt % PW/acidic Al_2_O_3_	85	4
14	30 wt % PW/Amberlyst^®^ 15	80	4.5
15	50 wt % H_3_PO_4_/Al_2_O_3_	74	5
16	30 wt % phosphomolybdic acid/Amberlyst^®^ 15	43	6

^a^Reaction carried out under neat conditions using 0.04 g/mmol of the catalyst and a **1a**/**2**/**3** 1:2:1 ratio. ^b^Yield of the pure isolated product. ^c^Activation by heating overnight at 100 °C. ^d^**1a**/**2**/**3** 1:2:2 ratio.

The Nafion^®^ catalysts showed the most promising results when carrying out the screenings. These catalysts were not further studied since they are no longer commercially available, and the preparation requires rather extreme conditions. The four catalysts chosen for further investigation according to the preliminary screenings shown in Table 1 were the activated resin Amberlyst^®^ 15 (Table 1, entry 6), 40 wt % PW on silica (Table 1, entry 9), a 30 wt % PW loading on montmorillonite K30 clay (Table 1, entry 11), and a 40 wt % PW loading on alumina (Table 1, entry 12).

**Figure 1:**
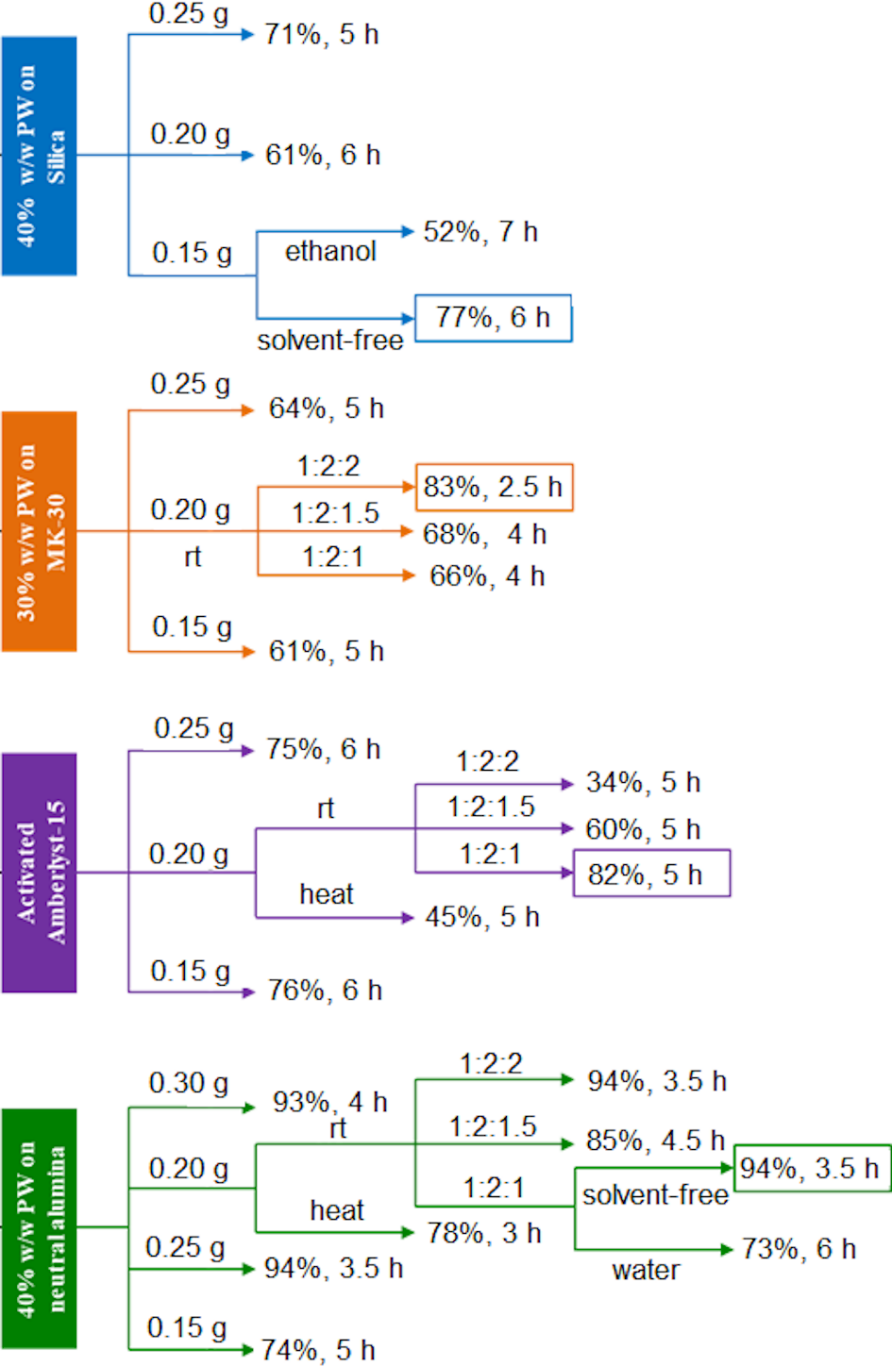
Optimization trials with the selected solid catalysts.

The study on the optimal reaction conditions shed a light on the acidity and the physical characteristics required for the reaction to be successful; the reaction requires strong acids. In addition, a peculiar result was obtained when analyzing the structure of the product obtained since the less frequently reported regioisomer, the unsymmetrical 1,2-DHP, was being obtained in a high yield and with a high selectivity. At this stage, further optimization and reaction trials were required in order to better understand the reaction conditions needed for the best results in terms of yield and selectivity of this transformation. The following investigation on each selected catalyst included changes in the molar ratio of the reagents, in the amount of catalyst, and in the temperature as well as the effect of the chosen green solvents (Figure 1).

Increasing the temperature did not significantly change the reaction yield or reduce the reaction time, while the presence of a green solvent, such as water or ethanol, negatively impacted the course of the reaction. Increasing the amount of catalyst did have a positive effect on the reaction, however, this was observed only up to a certain weight. The molar ratio of the reagents was altered by increasing the amount of ammonium acetate (**3**) in the model reaction. This again had no particularly positive effect. Since the presence of water was shown to be detrimental, the ammonium acetate (**3**) used was left to dry in a desiccator before use.

Reaction monitoring was mostly done using thin-layer chromatography (TLC) and at times also GC. These techniques showed the occurrence of at least three intermediates before reaching the ultimate product. No side products were observed at the end of all reactions, which shed a light on the selectivity obtained using the developed protocol. The results from the optimization trials are highlighted in Figure 1.

The optimal reaction conditions chosen included room temperature, a stoichiometric molar ratio of the reactants, using 40 wt % PW loaded on alumina under solvent-free conditions. These conditions satisfied the green protocol we were aiming for. Therefore, from this stage we moved onto the next one by changing the substrates to explore the versatility of the developed method.

The selectivity was promising even for the other substrates used (Table 2). Various substituted benzaldehydes were used, and all gave similar results. Deviations from the model reaction occurred in terms of the expected reaction time and yield, but generally, the deviations from the model reaction were minimal.

**Table 2:** Screening of different substrates.

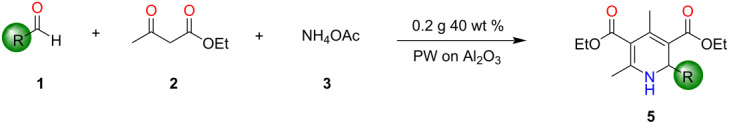			

product^a^	R	yield of **5** (%)^b^	time (h)

**5a**	C_6_H_5_ (**1a**)	94	4.5
**5b**	2-OH-C_6_H_4_ (**1b**)	73	3.5
**5c**	2,4-(OH)_2_-C_6_H_3_ (**1c**)	96	4
**5d**	2,4-Cl_2_-C_6_H_3_ (**1d**)	65	6
**5e**	3-CH_3_O-C_6_H_4_ (**1e**)	62	5
**5f**	2-CH_3_O-C_6_H_4_ (**1f**)	62	5.5
**5g**	4-CH_3_-C_6_H_4_ (**1g**)	64	4.5
**5h**	4-N(CH_3_)_2_-C_6_H_4_ (**1h**)	92	4
**5i**	naphthyl (**1i**)	90	4
**5j**	2,3-(methylenedioxy)-C_6_H_3_ (**1j**)	81	4.5

^a^The reactions were performed on a 5 mmol scale under neat conditions at room temperature and in the presence of 0.04 g/mmol 40 wt % PW on alumina at a molar ratio of 1:2:1. ^b^Pure isolated product.

Unexpectedly, when carrying out the reaction using aliphatic aldehydes under the same conditions, a different regioisomer, the commonly reported 1,4-DHP instead of the 1,2-DHP, was produced in the form of **4** with a high selectivity (Table 3), which was also reported by Cao and collaborators [19].

**Table 3:** Results obtained when using the aliphatic aldehydes **1** in the Hantzsch synthesis.

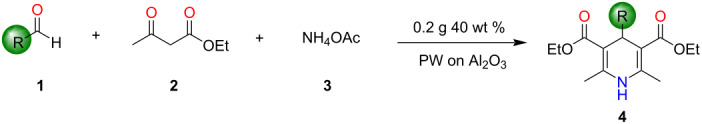			

product^a^	aldehyde	yield of **4** (%)^b^	time (h)

**4b**	cyclohexanal (**1k**)	82	4
**4c**	penten-2-al (**1l**)	79	4

^a^The reactions were performed on a 5 mmol scale under neat conditions at room temperature and in the presence of 0.04 g/mmol 40 wt % PW on alumina at a molar ratio of 1:2:1. ^b^Pure isolated product.

### Catalyst characterization and recyclability

The catalyst was analyzed by X-ray fluorescence (XRF) spectroscopy in order to ascertain the PW/Al_2_O_3_-support ratio. The mass percentage ratio of tungsten, which is the main component of the catalyst, and aluminium, the major element of the support, was used to determine the percentage of PW in the ensemble. According to the data obtained by XRF spectroscopy, the PW loading of 38.4 wt % was concordant to the theoretical value of 40 wt %.

**Figure 2:**
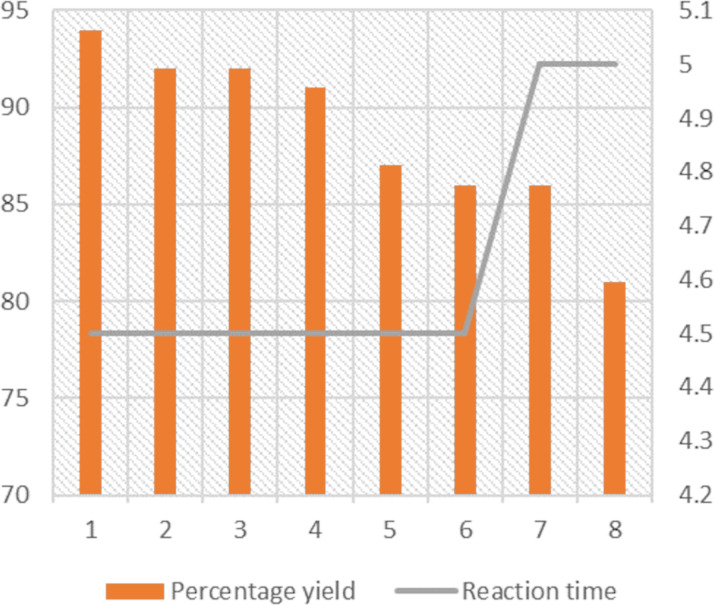
Graphical representation of the results obtained in the reusability test.

When the reusability test was carried out with the model reaction using the optimal catalyst, 40 wt % PW on alumina, a substantial yield loss of 13% was observed after the 8th cycle while the required reaction time increased by 30 minutes after the 7th cycle (Figure 2). This result confirmed the green character of the protocol, which is what we were aiming for.

### Green metrics

The green character of a reaction can be approximately quantified by calculating both the E-factor and the atom economy (AE), amongst other factors. The AE of the Hantzsch synthesis for the model reaction involving benzaldehyde (**1a**, 1 mol), ethyl acetoacetate (**2**, 2 mol) and ammonium acetate (**3**, 1 mol) is equal to 74%:

[1] $$\begin{array}{c} \text{AE}=\frac{m\text{ (product)}}{m\text{ (starting materials)}}\\ =\frac{\text{329}\text{.39 g}}{2\cdot 130.14\text{ g}+106.12\text{ g}+77.08\text{ g}}\cdot 100\%=74.3\% \end{array}$$

In order to take the amount of waste generated by the materials that are not directly involved in the reaction into consideration, the E-factor was also calculated:

[2] $$\begin{array}{c} \text{E}=\frac{m\text{ (product)}+m\text{ (catalyst)}}{m\text{ (catalyst)}+m\text{ (starting materials)}}\\ =\frac{\text{1}\text{.548 g + 0}\text{.2 g}}{\text{0}\text{.2 g}+\text{0}\text{.385 g}+\text{0}\text{.531 g}+\text{1}\text{.301 g}}=0.72 \end{array}$$

The mass used for the calculation is that of the starting materials of the model reaction and that of the catalyst used in the general procedure.

### Biological screening

The 1,4-DHP scaffold displays an extensive range of biological activities, including reversing multidrug resistance (through the inhibition of the P-glycoprotein) [26] and antiproliferative effects on human cancer cell lines [27]. We wondered whether the studied 1,2-DHPs could interfere with tumor cell growth. Thus, we selected a small subset of 1,2-DHPs and screened them against a panel of six human solid tumor cell lines. The results are shown in Table 4. Interestingly, the majority of the 1,2-DHPs displayed antiproliferative activity against all cell lines, in the low micromolar range. The most active compound was **5e**, which exhibited GI_50_ values in the range of 2.7–5.6 μM. The results obtained are comparable to those of the standard anticancer drug cisplatin (CDDP), which was used as reference drug.

**Table 4:** Antiproliferative activity (GI_50_ values) of selected 1,2-DHPs against human solid tumor cells.^a^

compound	cell line					
	A549	HBL-100	HeLa	SW1573	T-47D	WiDr

**5a**	29 ± 4.6	23 ± 1.8	21 ± 2.0	31 ± 0.7	21 ± 2.5	22 ± 2.4
**5b**	>100	>100	28 ± 7.5	>100	>100	>100
**5d**	14 ± 3.1	19 ± 3.0	12 ± 4.5	22 ± 1.2	17 ± 3.6	17 ± 1.2
**5e**	**5.1 ± 0.3**	**4.8 ± 0.4**	**2.7 ± 0.5**	**5.6 ± 1.0**	**4.0 ± 0.3**	**3.3 ± 0.5**
**5f**	43 ± 14	42 ± 6.2	26 ± 4.0	49 ± 12	33 ± 5.1	41 ± 9.6
**5g**	18 ± 4.9	20 ± 0.9	22 ± 6.6	18 ± 6.8	22 ± 2.1	18 ± 1.6
**5h**	34 ± 4.7	38 ± 4.0	28 ± 4.6	33 ± 6.1	31 ± 0.9	39 ± 8.9
**5i**	23 ± 6.8	26 ± 2.0	18 ± 5.0	30 ± 0.3	20 ± 2.0	33 ± 4.8
**5j**	16 ± 6.7	16 ± 3.5	**4.9 ± 1.0**	11 ± 1.7	15 ± 1.9	19 ± 3.3
CDDP	**4.9 ± 0.2**	**1.9 ± 0.2**	**1.8 ± 0.5**	**2.7 ± 0.4**	17 ± 3.3	23 ± 4.3

^a^GI_50_ values are given in µM. The standard deviation was calculated from at least two independent experiments. CDDP (cisplatin) was used as a reference compound. Values in bold face represent the best antiproliferative data against tumor cell lines (GI_50_ < 10 µM).

## Conclusion

The one-pot multicomponent Hantzsch reaction for the synthesis of substituted dihydropyridines was performed under green heterogeneous and neat conditions in the presence of 0.04 g/mmol of a 40 wt % phosphotungstic acid on alumina catalyst, which is simple, safe and environmentally benign to prepare, fully recoverable, and reusable for up to 8 runs. A high AE of 74% and a low E-factor of 0.72 highlight the green character of the procedure. More importantly, PW/alumina was able to catalyze a wide range of reactions involving different aromatic aldehydes to give products in good to excellent yields and interestingly all with the same general structure, corresponding to the 1,2-DHP regioisomer, unless when using aliphatic aldehydes.

## Experimental

### General

All the chemicals used were purchased form Sigma-Aldrich. IR spectroscopy studies were conducted on a Shimadzu IRAffinity-1 FTIR spectrometer calibrated against 1602 cm^−1^ polystyrene absorbance spectra. The ^1^H NMR and ^13^C NMR spectra were measured on a Bruker Avance III HD^®^ NMR spectrometer equipped with an Ascend 500 11.75 Tesla superconducting magnet, operating at 500.13 MHz for ^1^H and 125.76 MHz for ^13^C, and a multinuclear 5 mm PABBO probe. Melting points were recorded on a Stuart^®^ SMP11 melting point apparatus. Reactions were monitored by TLC and GC. Mass spectra were measured via a Thermo Scientific GC/MS DSQ II device, which contained a column: EC-5 30 m × 0.25 mm i.d. × 0.25 µm or using the direct-infusion method using a Waters^®^ ACQUITY^®^ TQD system with a tandem quadrupole mass spectrometer. The software used was ThermoXcalibur 2.2 SP1.48. The XRF spectroscopy analysis of the catalyst was performed using a Bruker S2 Ranger^®^.

### Catalyst preparation

The method previously reported by Zhu et al*.* was used to prepare several supported heteropoly acids (silicotungstic and PW) on various supports at different loadings [28]. For 1 g of a catalyst batch with a loading of 20 wt %, 0.8 g of the support and 0.2 g of a heteropoly acid were stirred in a minimum amount of distilled water to form a slurry for 8 hours at room temperature in a 10 mL round-bottomed flask. Then, the catalyst was dried overnight at 110 °C and ultimately calcined at 250 °C in a furnace under air for 4 h to obtain a white powder (1 g), which was stored in a calcium chloride/silica-filled desiccator.

### General method

The general method involved the addition of 1 equiv of the aldehyde (5 mmol), 2 equiv ethyl acetoacetate (**2**, 10 mmol), and 1 equiv ammonium acetate (**3**, 5 mmol) in one vessel. The reactants were left to stir together with 0.2 g of the catalyst (40 wt % PW on alumina). At intervals of 30 minutes, the reaction mixture was analyzed using TLC, GC, or both. With time, the reaction mixture was observed to change from colorless to yellow, which darkened or brightened to orange or yellow and thickened with the occurrence of crystals on the sides of the flask. Once the spot or the peak corresponding to the benzaldehyde disappeared on the TLC plate or in the gas chromatogram, the time was taken as the reaction was finished. The reaction mixture, obtained as a viscous oil, was filtered through a sinter funnel to remove any catalyst and washed using acetone, which was then evaporated in a rotary evaporator. The resultant crystals were then recrystallized using hot ethanol. The products were then characterized via IR, NMR, and MS analysis.

### Hot filtration test

The optimized model reaction was monitored by GC, and the catalyst was left in the reaction mixture for 30 minutes in order to confirm heterogeneity. During these 30 minutes, the reaction started. However, upon removal of the catalyst by filtration, the reaction was left to carry on but stopped, and therefore catalyst leaching was not evident.

### Antiproliferative tests

We selected the cancer cell lines A549 and SW1573 (nonsmall-cell lung), HBL-100, as well as T-47D (breast), HeLa (cervix), and WiDr (colon) to evaluate the antiproliferative activity. The tests were performed in 96-well plates using the SRB assay [29] with the following specifications: the cell seeding density was 2500 cells/well for A549, HBL-100, HeLa, and SW1573, and 5000 cells/well for T-47D and WiDr. The drug incubation time was 48 h. The optical density of each well was measured at 530 (primary) and 620 nm (secondary). The antiproliferative activity, expressed as GI_50_ values, was calculated according to the NCI formulas [30].

## Supporting Information

File 1 Analytical data of the products.

## References

[1] Dömling A, AlQahtani A D, Zhu J, Wang Q, Wang M-X (2014). General Introduction to MCRs: Past, Present, and Future. Multicomponent Reactions in Organic Synthesis.

[2] Ganem B (2009). Acc Chem Res.

[3] Alvim H G O, da Silva Júnior E N, Neto B A D (2014). RSC Adv.

[4] Touré B B, Hall D G (2009). Chem Rev.

[5] Sheldon R A (2017). Green Chem.

[6] Ciriminna R, Pagliaro M (2013). Org Process Res Dev.

[7] Abdel-Mohsen H T, Conrad J, Beifuss U (2012). Green Chem.

[8] Ghorbani-Choghamarani A, Zolfigol M A, Salehi P, Ghaemi E, Madrakian E, Nasr-Isfahani H, Shahamirian M (2008). Acta Chim Slov.

[9] Sharma V K, Singh S K (2017). RSC Adv.

[10] Javanbakht S, Shaabani A (2020). ACS Appl Bio Mater.

[11] Lavilla R (2002). J Chem Soc, Perkin Trans 1.

[12] Silva E M P, Varandas P A M M, Silva A M S (2013). Synthesis.

[13] Nasr-Esfahani M, Montazerozohori M, Raeatikia R (2014). Maejo Int J Sci Technol.

[14] Pal S, Choudhury L H, Parvin T (2013). Synth Commun.

[15] Pacheco S R, Braga T C, da Silva D L, Horta L P, Reis F S, Ruiz A L T G, de Carvalho J E, Modolo L V, de Fatima A (2013). Med Chem.

[16] Pramanik A, Saha M, Bhar S (2012). ISRN Org Chem.

[17] Vanden Eynde J, Mayence A (2003). Molecules.

[18] D'Alessandro O, Sathicq Á G, Sambeth J E, Thomas H J, Romanelli G P (2015). Catal Commun.

[19] Shen L, Cao S, Wu J, Zhang J, Li H, Liu N, Qian X (2009). Green Chem.

[20] Bosica G, Abdilla R (2017). Green Chem.

[21] Sheldon R A (2008). Chem Commun.

[22] Cave G W V, Raston C L, Scott J L (2001). Chem Commun.

[23] Heravi M M, Bakhtiari K, Javadi N M, Bamoharram F F, Saeedi M, Oskooie H A (2007). J Mol Catal A: Chem.

[24] Bitaraf M, Amoozadeh A, Otokesh S (2016). J Chin Chem Soc.

[25] Liu Y, Zhao G, Wang D, Li Y (2015). Natl Sci Rev.

[26] Firuzi O, Javidnia K, Mansourabadi E, Saso L, Mehdipour A R, Miri R (2013). Arch Pharmacal Res.

[27] Sharma M G, Vala R M, Patel D M, Lagunes I, Fernandes M X, Padrón J M, Ramkumar V, Gardas R L, Patel H M (2018). ChemistrySelect.

[28] Zhu S, Zhu Y, Gao X, Mo T, Zhu Y, Li Y (2013). Bioresour Technol.

[29] Orellana E A, Kasinski A L (2016). Bio-Protoc.

[30] Monks A, Scudiero D, Skehan P, Shoemaker R, Paull K, Vistica D, Hose C, Langley J, Cronise P, Vaigro-Wolff A (1991). J Natl Cancer Inst.

