# Correction: One-pot multicomponent green Hantzsch synthesis of 1,2-dihydropyridine derivatives with antiproliferative activity

**DOI:** 10.3762/bjoc.17.130

**Published:** 2021-08-10

**Authors:** Giovanna Bosica, Kaylie Demanuele, José Manuel Padrón, Adrián Puerta

**Affiliations:** 1 Department of Chemistry, University of Malta, Msida, MSD 2080 Malta; 2 BioLab, Instituto Universitario de Bio-Orgánica “Antonio González” (IUBO-AG), Universidad de La Laguna, c/Astrofísico Francisco Sánchez 2, 38206 La Laguna, Spain

**Keywords:** antiproliferative activity, 1,2-dihydropyridines, green Hantzsch synthesis, heterogeneous catalysis, one-pot multicomponent reaction

The authors noticed that the E-factor (E) was calculated using a wrong Equation 2 in the original publication:


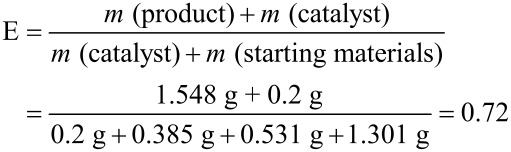


The correct Equation 2 should be the following:


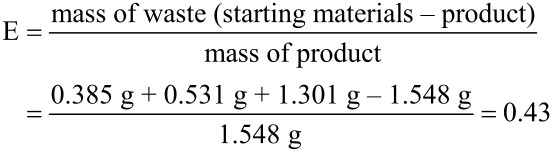


Accordingly, the Conclusion section should be amended with the following sentence:

A high AE of 74% and a low E-factor of 0.43 highlight the green character of the procedure.

The authors apologize for any inconvenience caused by the originally published wrong Equation 2.

