# Supplementary material for: One-pot multicomponent green Hantzsch synthesis of 1,2-dihydropyridine derivatives with antiproliferative activity
Source: Beilstein J Org Chem. 2020 Nov 24;16:2862–9. doi: 10.3762/bjoc.16.235 (PMC7705860; doi:10.3762/bjoc.16.235)
Supplement: File 1 — Analytical data of the products. [file Beilstein_J_Org_Chem-16-2862-s001.pdf]

BEILSTEIN JOURNAL OF ORGANIC CHEMISTRY

Supporting Information

for

One-pot multicomponent green Hantzsch synthesis of 1,2-dihydropyridine derivatives with antiproliferative activity

Giovanna Bosica, Kaylie Demanuele, José M. Padrón and Adrián Puerta

Beilstein J. Org. Chem. **2020,** *16,* 2862–2869. doi:10.3762/bjoc.16.235

Analytical data of the products

License and Terms: This is a supporting information file under the terms of the Creative Commons Attribution License (https://creativecommons.org/licenses/by/4.0). Please note that the reuse, redistribution and reproduction in particular requires that the authors and source are credited.
The license is subject to the *Beilstein Journal of Organic Chemistry* terms and conditions: (https://www.beilstein-journals.org/bjoc)

Analytical data of products

Diethyl 4,6-dimethyl-2-phenyl-1,2-dihydropyridine-3,5-dicarboxylate (**5a**) [1,2]. $C_{19}H_{23}NO_4$**.** Yellow solid. M.P.: 145 °C. IR (KBr, cm^{-1}): 3429 (m), 3327 (w), 3018 (s), 2939 (m), 2899 (m), 1685 (s), 1701 (s), 1651 (m), 1444 (m), 1371 (s),1222 (s). ^{1}H NMR (500 MHz, Chloroform-*d*) δ ppm= 7.37 – 7.19 (m, 5H), 5.63 (d, *J* = 4.0 Hz, 1H), 5.27 (s, 1H), 4.31 – 4.06 (m, 4H), 2.41 (s, 3H), 2.25 (s, 3H), 1.33 (t, *J* = 7.1 Hz, 3H), 1.24 (t, *J* = 7.0 Hz, 3H). ^{13}C NMR (126 MHz, $CDCl_3$, 298 K) δ (ppm) = 167.85, 166.88, 154.23, 146.31, 143.27, 128.59, 127.77, 126.40, 111.09, 103.91, 59.90, 59.64, 55.12, 21.31, 19.52, 14.36, 14.25. MS (ES+): m/z =330.25 (M+1), 329.23 (M), 328.21, 315.22, 314.16, 298.20, 252.14, 224.14, 196.12, 178.05, 150.09, 115.04, 77.03.

Diethyl 2-(2-hydroxyphenyl)-4,6-dimethyl-1,2-dihydropyridine-3,5-dicarboxylate (**5b**) [3]. $C_{19}H_{23}NO_5$. Yellow solid. M.P.: 91 °C. IR (KBr, cm^{-1}): 3333 (w, br), 3014 (w), 2958 (w), 1634 (m), 1612 (m), 1488 (m), 1215 (s), 668 (m). ^{1}H NMR (500 MHz, Chloroform-*d*) δ (ppm) = 8.62 (dd, *J* = 7.9, 1.7 Hz, 1H), 7.57 (ddd, *J* = 8.3, 7.2, 1.7 Hz, 1H), 7.41 – 7.31 (m, 2H), 4.49 (p, *J* = 7.1 Hz, 2H), 4.11 – 4.06 (m, 4H), 2.81 (s, 3H), 2.69 (s, 3H), 1.44 (t, *J* = 7.2 Hz, 3H), 1.15 (t, *J*=7.1 Hz, 3 H). ^{13}C NMR (126 MHz, Acetone) δ (ppm) = 168.41, 161.06, 160.24, 153.76, 152.96, 150.37, 133.44, 126.22, 125.39, 119.95, 117.37, 115.01, 62.76, 58.86, 53.92, 23.89, 19.47, 14.49, 14.48. MS (ES+): m/z= 345.23 (M), 297.09, 268.07, 248.0, 225.13, 152.09, 139.07, 126.06, 115.07, 77.05.

Diethyl 2-(2,4-dihydroxyphenyl)-4,6-dimethyl-1,2-dihydropyridine-3,5-dicarboxylate (**5c**). $C_{19}H_{23}NO_6$. Yellow solid. M.P.: 118 °C. IR (KBr, cm^{-1}): 3335 (m, br), 3014 (w), 2959 (w), 1636 (m), 1614 (m), 1488 (m), 1215 (s), 669 (m). ^{1}H NMR (500 MHz, Chloroform-*d*) δ (ppm) = 8.49 (d, *J* = 8.7 Hz, 1H), 6.88 (dd, *J* = 8.7, 2.4 Hz, 2H), 6.83

(d, *J* = 2.4 Hz, 1H), 4.50 (q, *J* = 7.1 Hz, 4H), 4.29 – 4.18 (m, 2H), 2.80 (s, 3H), 2.68 (s, 3H), 1.46 (t, *J* = 7.1 Hz, 6H), 1.37 – 1.27 (m, 1H), 1.30 – 1.24 (m, 1H). ^{13}C NMR (126 MHz, $CDCl_3$) δ (ppm) = 171.63, 167.50, 166.09, 153.62, 148.74, 136.76, 134.42, 132.95, 129.54, 127.45, 107.80, 104.15, 60.05, 59.65, 51.22, 21.10, 19.21, 14.45, 14.31. MS (ES+): m/z = 361.46, (M), 359.35, 356.91.

Diethyl 2-(2,4-dichlorophenyl)-4,6-dimethyl-1,2-dihydropyridine-3,5-dicarboxylate (**5d**). $C_{19}H_{21}Cl_2NO_4$. Yellow solid. M.P.: 82-84 °C IR (KBr, cm^{-1}): 3018 (w) 2956 (w), 2357(w), 2328 (m), 1606 (m), 1219 (m), 771 (s), 667 (m). ^{1}H NMR (500 MHz, Chloroform-*d*) δ (ppm) = 7.41 (d, *J* = 2.1 Hz, 1H), 7.28 – 7.19 (m, 2H), 5.96 (d, *J* = 4.2 Hz, 1H), 5.56 (d, *J* = 4.2 Hz, 1H), 4.30 – 4.04 (m, 4H), 2.52 (s, 3H), 2.15 (s, 3H), 1.33 (t, *J* = 7.1 Hz, 3H), 1.16 (t, *J* = 7.1 Hz, 3H). ^{13}C NMR (126 MHz, $CDCl_3$) δ (ppm) = 171.72, 167.48, 166.09, 153.83, 148.71, 136.71, 134.31, 132.85, 129.53, 127.45, 107.81, 104.10, 60.00, 59.75, 51.22, 21.16, 19.31, 14.36, 14.21. MS (ES+): m/z = 398.3, 397.10 (M-1), 368.03, 352.05, 252.13, 224.11, 196.07.

Diethyl 2-(3-methoxyphenyl)-4,6-dimethyl-1,2-dihydropyridine-3,5-dicarboxylate (**5e**) [2]. $C_{20}H_{25}NO_5$ Yellow solid. M.P.: 119-121 °C. IR (KBr, cm^{-1}): 3333 (w, br), 3014 (w), 2958 (w), 1633 (m), 1612 (m), 1489 (m), 1215 (s), 667 (m). H NMR (500 MHz, Chloroform-*d*) δ (ppm)= 7.23 (t, *J* = 7.8 Hz, 1H), 6.94 – 6.88 (m, 2H), 6.81 (ddd, *J* = 8.2, 2.6, 1.0 Hz, 1H), 5.61 (d, *J* = 4.1 Hz, 1H), 5.29 (d, *J* = 3.8 Hz, 1H), 4.24 (dq, *J* = 10.8, 7.1 Hz, 1H), 4.17 (dq, *J* = 7.2, 3.4 Hz, 3H), 3.79 (s, 3H), 2.41 (s, 3H), 2.25 (s, 3H), 1.32 (t, *J* = 7.1 Hz, 3H), 1.25 (t, *J* = 7.1 Hz, 3H). ^{13}C NMR (126 MHz, $CDCl_3$, 298 K) δ (ppm) = 14.45, 14.48, 19.63, 21.33, 55.12, 55.24, 59.74, 60.05, 103.93, 110.7,

112.24, 113.2, 118.81, 129.72, 144.92, 146.26, 154.23, 159.88, 167.00, 167.95. MS (ES+): m/z =359.73, 355.15, 252.17, 224.13, 196.11, 178.08.

Diethyl 2-(2-methoxyphenyl)-4,6-dimethyl-1,2-dihydropyridine-3,5-dicarboxylate (**5f**) [2]. $C_{20}H_{25}NO_5$ Yellow solid. M.P.: 124 °C. IR (KBr, cm^{-1}): 3427 (m), 3325 (w), 3018 (s), 2981(m), 1681 (s), 1648 (s), 1498 (m), 1215 (s), 750 (s). ^1H NMR (500 MHz, $CDCl_3$, 298 K) δ (ppm) = 7.28 - 7.23 (m, 1H), 7.18 (dd, *J* = 1.7, 7.5 Hz, 1H), 6.94 - 6.89 (m, 2H), 5.94 (d, *J* = 3.8 Hz, 1H), 5.73 (br s, 1H), 4.25 - 4.05 (m, 4H), 3.93 (s, 3H), 2.50 (s, 3H), 2.14 (s, 3H), 1.31 (t, *J* = 7.1 Hz, 3H), 1.19 (t, *J* = 7.1 Hz, 3H). ^{13}C NMR (126 MHz, $CDCl_3$) δ (ppm) = 167.81, 166.90, 156.68, 154. 99, 143.86, 129.01, 128.77, 128.01, 120.55, 110.36, 107.8,–102.87, 59.74, 59.39, 55.45, 49.42, 21.51, 19.65, 14.37, 14.28. MS (ES+): m/z =359.23, 330.16, 252.13, 224.13, 196.08, 178.07, 150.09.

Diethyl 2-(4-methylphenyl)-4,6-dimethyl-1,2-dihydropyridine-3,5-dicarboxylate (**5g**) [2]. $C_{20}H_{25}NO_4$ Yellow solid. M.P.: 108 °C. IR (KBr, cm^{-1}): 3333 (w, br), 3014 (w), 2958 (w), 1633 (m), 1612 (m), 1489 (m), 1215 (s), 667 (m). ^1H NMR (500 MHz, $CDCl_3$, 298 K) δ (ppm) = 7.14-7.16 (m, 2H), 6.66-6.63, (m, 2H), 5.55 (s, 1H), 4.17-4.07 (m, 4H), 2.82 (s, 1H), 2.4 (s, 3H), 2.25 (s, 3H) 2.07 (s, 3H), 1.27 (t, *J*= 7.0 Hz, 3H), 1.21 (t, *J*= 7.0 Hz, 3H). ^{13}C NMR (126 MHz, $CDCl_3$) δ (ppm) = 167.89, 166.94, 154.18, 145.95, 140.42, 137.45, 129.25, 129.13, 126.36, 110.76, 103.46, 59.86, 59.59, 54.82, 21.33, 21.10, 19.53, 14.36, 14.30. MS (ES+): m/z = 343.24 (M), 314.20, 270.20, 252.17, 224.13, 196.11, 178.08.

Diethyl 2-(4-dimethylaminophenyl)-4,6-dimethyl-1,2-dihydropyridine-3,5-dicarboxylate (**5h**). $C_{21}H_{28}N_2O_4$ Yellow solid. M.P.: 128 °C. IR (KBr, cm^{-1}): 3018 (w) 2954 (w), 2357(w), 2325 (m), 1603 (m), 1219 (m), 772 (s), 665 (m). ^{1}H NMR (500 MHz, $CDCl_3$, 298 K) δ (ppm) = 7.22 (d, *J* = 8.5 Hz, 2H), 6.67 (d, *J* = 7.9 Hz, 2H), 5.50 (d, *J* = 4.5 Hz, 1H), 5.23 (br s, 1H), 4.27 - 4.10 (m, 4H), 2.94 (s, 6H), 2.40 (s, 3H), 2.22 (s, 3H), 1.33 (t, *J*=7.03 Hz, 3 H), 1.23 (t, *J*=7.09 Hz, 3 H). ^{13}C NMR (126 MHz, $CDCl_3$) δ (ppm) = 168.13, 167.15, 154.27, 150.33, 145.54, 131.73, 127.57, 112.53, 111.24, 103.08, 59.87, 59.62, 54.84, 40.64, 21.55, 19.68, 14.50, 14.42. MS (ES+): m/z = 372.46 (M), 357.23, 299.22, 271.20, 253.18, 224.15, 196.11, 121.13, 105.08, 91.07.

Diethyl 4,6-dimethyl-2-(naphthalen-1-yl)-1,2-dihydropyridine-3,5-dicarboxylate (**5i**). $C_{23}H_{25}NO_4$. M.P.: 138-139 °C. IR (KBr cm^{-1}): 3432, (m), 3332 (m), 3018 (s), 2872 (m), 1672 (m), 1615 (m), 1215 (s), 757 (s). ^{1}H NMR (500 MHz, CHLOROFORM-d) δ (ppm) = 8.21 (d, *J*=8.4 Hz, 1 H), 7.92 (d, *J*=8.1 Hz, 1 H), 7.81 (d, *J*=8.1 Hz, 1 H), 7.45 - 7.67 (m, 4 H), 6.52 (d, *J*=3.5 Hz, 1 H), 5.21 (br s, 1 H), 4.14 - 4.30 (m, 2 H), 4.02 (qq, *J*=10.7, 7.11 Hz, 2 H), 2.52 - 2.57 (m, 3 H), 2.07 (s, 3 H), 1.35 (t, *J*=7.2 Hz, 3 H), 1.04 (t, *J*=7.1 Hz, 3 H). ^{13}C NMR (126 MHz, Acetone) δ (ppm) = 168.04, 167.93, 157.36, 155.73, 147.93, 138.66, 135.29, 132.62, 129.69, 128.96, 127.01, 126.91, 125.76, 124.27, 109.71, 103.01, 62.54, 59.77, 51.33, 23.18, 20.83, 14.82, 13.61. MS (ES+): m/z= 379.24, 350.19, 306.18, 278.17, 252.14, 196.06, 178.10, 128.11, 107.11, 77.04.

Diethyl 2-(benzo[*d*][1,3]dioxol-4-yl)-4,6-dimethyl-1,2-dihydropyridine-3,5-dicarboxylate (**5j**). $C_{20}H_{23}NO_6$. M.P.: 142 °C. IR (KBr, cm^{-1}): 3429 (m), 3327 (w), 3018 (s), 2981(m), 2899 (m), 1683 (s), 1652 (s), 1502 (m), 1215 (s), 752 (s), 665 (m). ^{1}H NMR (500 MHz, DMSO-d_6) δ (ppm) = 8.58 (d, *J* = 4.6 Hz, 1H), 6.83 (d, *J* = 8.0 Hz,

1H), 6.74 (d, *J* = 1.8 Hz, 1H), 6.69 – 6.63 (m, 1H), 5.97 (s, 2H), 5.41 (d, *J* = 4.4 Hz, 1H), 4.15 – 3.97 (m, 4H), 2.31 (s, 3H), 2.18 (s, 3H), 1.19 (dt, *J* = 19.0, 7.1 Hz, 3H) 1.15 (t, *J* = 19.0, 7.1 Hz, 3H). ^{13}C NMR (126 MHz, $CDCl_3$, 298 K) δ (ppm) = 167.98, 167.01, 154.03, 147.95, 147.29, 146.02, 137.68, 119.83, 110.89, 108.22, 107.27, 103.49, 101.12, 60.05, 59.78, 55.07, 21.40, 19.67, 14.47, 14.43. MS (ES+): m/z 373.4 (M), 358.21, 344.04, 300.3, 252.2, 224.06, 196.12.

Diethyl 4-cyclohexyl-2,6-dimethyl-1,4-dihydropyridine-3,5-dicarboxylate (**4b**) [4]. $C_{19}H_{29}NO_4$. M.P: 111-114 °C. IR (KBr, cm^{-1}): 3433 (m), 3335 (m), 2872 (m), 1674 (m), 1622 (m), 1215 (m), 1099 (w), 756 (s). ^{1}H NMR (500 MHz, $CDCl_3$) δ (ppm) = 5.53 (s, 1H), 4.26 – 4.10 (m, 4H), 3.92 (d, *J* = 5.7 Hz, 1H), 2.32 (s, 6H), 1.60 (d, *J* = 46.9 Hz, 2H), 1.58 (br s, 4H), 1.32 (t, *J* = 7.1 Hz, 6H), 1.12-1.08 (m, 3H), 0.98-0.93 (m, 2H). MS (ES+): m/z =335.4, 252.14, 224.14, 196.07, 179.16.

(*E*)-Diethyl 4-(but-1-en-1-yl)-2,6-dimethyl-1,4-dihydropyridine-3,5-dicarboxylate (**4c**). $C_{17}H_{25}NO_4$. Yellow solid. M.P: 125-128 °C. IR (KBr, cm^{-1}): 3435 (m), 3342 (m), 3016 (m), 2960 (m), 1678 (m), 1465 (m), 1273 (m), 1215 (s), 1099 (w), 754 (s). ^{1}H NMR (500 MHz, $CDCl_3$) δ (ppm) = 5.41 (br s, 1H), 5.33 (t, *J* = 4.4 Hz, 2H), 4.38 (s, 1H), 4.22 (dd, *J* = 10.8, 7.1 Hz, 3H), 4.13 (dd, *J* = 10.8, 7.1 Hz, 1H), 2.29 (s, 6H), 1.95 (dd, *J* = 7.4, 4.2 Hz, 2H), 1.28 (t, *J* = 7.1 Hz, 6H), 0.91 (t, *J* = 7.4 Hz, 3H).^{13}C NMR (126 MHz, $CDCl_3$) δ (ppm) = 167.84, 144.25, 130.57, 102.32, 59.60, 36.12, 25.34, 19.44, 14.41, 13.82. MS (ES+): m/z = 308.23, 307.21, 306.20, 305.20.

References

1. Shen, L.; Cao, S.; Wu, J.; Zhang, J.; Li, H.; Liu, N.; Qian, X. *Green Chem.* **2009**, *11*, 1414-1420.

2. O'Callaghan, Conor N. *Journal of Chemical Research, Miniprint*, **1986**, *9*, 2901 – 2915.

3. O'Callaghan, Conor N. *Synthesis*, **1987**, *5*, 499 – 503.

4. Xie, Shasha; Li, Defang; Huang, Hanchu; Zhang, Fuyuan; Chen, Yiyun. *J. Am. Chem. Soc.*, **2019**, *141*, 16237 – 16242.
